# 

**Published:** 1980

**Authors:** 


Contents

3 **Editorial**

4 **Small Bowel Volvulus – The Commonest Abdominal Emergency in Nepal**
I. O. McDonald and D. B. G. Hawkin

9 **Patients and Spiders**
Philip Radford

11 **Some Aspects of Eye Disease in the Transkei**
J. D. Huggan

12 **Obituaries**

Professor Geoffrey Dixon

Dr. Kathleen Iles

Mr. R. G. Paul

16 **Abstracts**
South West Surgical Club Meeting, Bristol, 31st October 1980

21 **Book Reviews**
Surgical Problems in the Aged, K. D. J. Vowles
Analysis of Primary Medical Care, W. J. Stephen

22 **Examination Results**

